# Myolipoma Affecting the Erector Spinae: A Case Report in a Child

**DOI:** 10.1155/2009/520126

**Published:** 2009-11-08

**Authors:** M. T. R. Parratt, K. Gokaraju, B. G. I. Spiegelberg, J. Miles, S. R. Cannon, T. W. R. Briggs

**Affiliations:** The Sarcoma Unit, Royal National Orthopaedic Hospital, Brockley Hill, Stanmore HA7 4LP, UK

## Abstract

Myolipoma is a rare, benign, lipomatous tumour which most commonly occurs in the retroperitoneum, pelvis, and abdomen. A 4-year-old boy presented with a painless enlarging mass in the left paraspinal region. Magnetic Resonance Imaging (MRI) revealed a soft tissue lesion with high fat content and areas of calcification. Excision and histopathological analysis revealed a tumour composed of lobules of mature adipose tissue and broad septa of well-differentiated smooth muscle tissue. The smooth muscle nature of the nonfatty component was demonstrated by a diffuse and strong immunoreactivity for smooth muscle actin and desmin. The mass was reported as a myolipoma. The patient made an unremarkable recovery from surgery and remains healthy with no signs of recurrence at seven years. This paper represents the youngest patient diagnosed with this rare soft tissue tumour which is normally confined to the adult population. A newly reported site of the tumour is also highlighted.

## 1. Introduction

Myolipoma is an extremely rare benign tumour related to lipoma. It is composed of a mixed proliferation of adipose and smooth muscle tissues presenting, in most patients, as a large painless mass. The tumour is benign with no reported cases of recurrence or metastasis, suggesting that complete surgical excision is curative. Most cases of this tumour have been reported in the retroperitoneum, pelvis, and abdomen. There are fewer reports of the tumour affecting other locations. We present a case of myolipoma affecting the erector spinae muscle group. As far as we are aware, this is the first reported case of the tumour in this location. Furthermore, this case represents the youngest patient diagnosed with a myolipoma.

## 2. Case History

**Figure 1:**
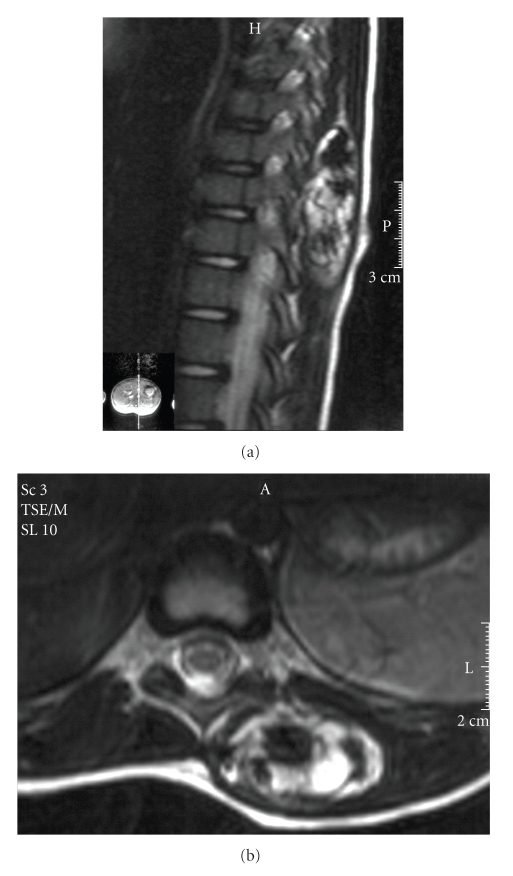
Sagittal and axial T2 weighted MRI images demonstrating a well-defined paraspinal mass.

**Figure 2:**
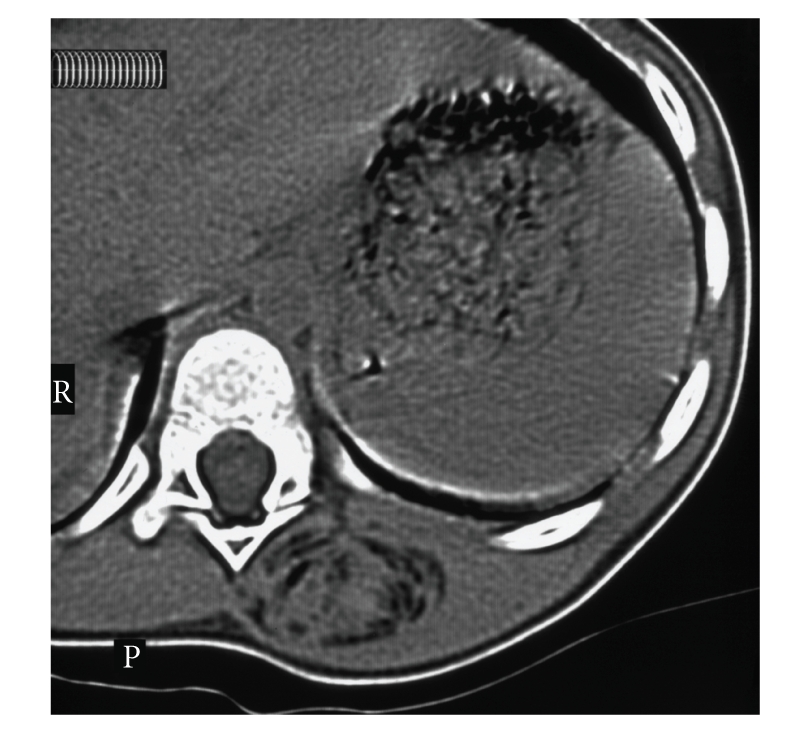
Axial CT image demonstrating a paraspinal mass composed of a mixture of fat and muscle. No bony involvement noted.

A four-year-old male was referred to our institution with a two-year history of a painless swelling on the left side of the back. The mass had been slowly increasing in size with the child's growth. The patient was otherwise healthy. Examination revealed a firm nonfluctuant, nonpulsatile mass in the left paraspinal region. No tethering or overlying skin changes were noted. Further examination was unremarkable. Investigations included ultrasound, computed tomography, and MRI. The ultrasound identified a tense cystic paraspinal mass. Magnetic resonance imaging (MRI) was performed and demonstrated a well-defined intramuscular mass overlying the lower thoracic spine. The mass was of fat signal intensity with multiple large areas of low signal intensity in keeping with calcification. No involvement of the posterior spinal elements or intraspinal extension was noted (Figures 1(a) and 1(b)). A CT scan was also performed and demonstrated a lesion composed of fat and other soft tissue (Figure 2). There was some peripheral mineralisation. All imaging investigations pointed towards a provisional diagnosis of a tumour with very high fat content which, in this age group, could be lipofibromatosis, fibrolipoma, or lipoblastoma.

**Figure 3:**
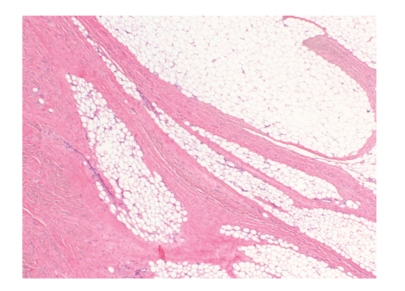
Low magnification view of myolipoma. The tumour is characterised by lobules of adult-type (mature) adipose tissue intimately admixed with sheets and septa of smooth muscle. Both components lack cellular atypia (H&E stain; 2× magnification).

**Figure 4:**
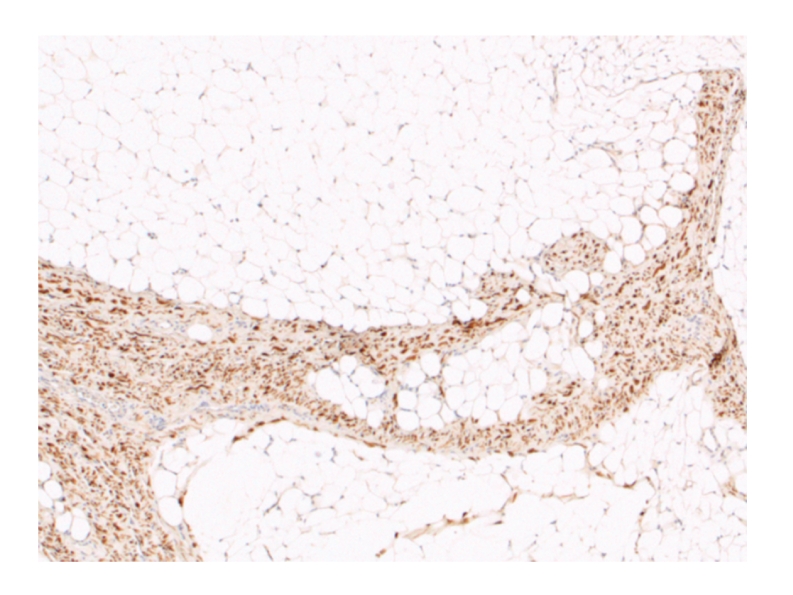
Immunohistochemistry. Picture showing immunoreactivity for desmin, a myogenic marker, in the nonfatty component.

The patient attended for excision biopsy and an ovoid tumour (6.3 × 3.9 × 2.6 cm) was resected. Macroscopically, the mass appeared covered by a thin layer of skeletal muscle. Histologically, the tumour was composed of lobules of adult-type mature adipose tissue separated by broad septa of well-differentiated smooth muscle tissue. This was characterised by elongated spindle cells with cigar-shaped nuclei and eosinophilic cytoplasms, arranged with a fascicular growth pattern (Figure 3). Both components (fatty and smooth muscle) showed no evidence of cellular atypia, mitoses, or necrosis. The smooth muscle nature of the nonfatty component was demonstrated by a diffuse and strong immunoreactivity for smooth muscle actin and desmin (Figure 4). S100 protein and CD34 were negative in the smooth muscle component. A diagnosis of lipoma with smooth muscle differentiation (myolipoma) was made.

The patient made an unremarkable recovery from surgery and remains healthy with no signs of recurrence.

## 3. Discussion

Myolipoma was first described by Meis and Enzinger in 1991 [1], who presented a series of nine patients with a previously undescribed benign soft tissue tumour. The tumour was composed of variable amounts of benign smooth muscle and mature adipose tissue. Three tumours were found in the retroperitoneum, two in the inguinal region, two in the abdominal cavity, and one each in the rectus sheath and subcutaneous tissue. This series remains the largest in the literature. It is a rare lesion; only 22 cases have been reported, with varying anatomical locations. However, it may have been reported as “fibrolipoleiomyoma” or “lipoleiomyoma,” particularly in cases of uterine tumours. The tumour most commonly occurs in the pelvis, abdomen, and retroperitoneum. Rarer sites include the eyelid [2], orbit [3], pericardium [4], and the tongue base (as a presentation of Gorlin's syndrome [5]). Intradural myolipoma has also been reported and can be associated with the tethered cord syndrome [6, 7].

Presentation tends to be with an enlarging painless mass. Most reported cases describe large tumours averaging 16 cm in the greatest dimension [1] and with a diameter of at least 9 cm [8]. It is most prevalent in the 5th and 6th decades of life, with a slight predilection for women [9]. The youngest patient reported, prior to this case, was 12 years old [7], all other reports being in adults.

There is paucity of information regarding the imaging of the lesion. Ultrasound may demonstrate a heterogeneous mass [10]. In uterine tumours, a hypoechoic ring (of myometrium) may be exhibited allowing preoperative diagnosis of myolipoma, which has been confirmed by histopathological evaluation [11, 12]. CT may be helpful in defining whether there is any bony involvement of the tumour, as in this case. Also, it may demonstrate images identical to subcutaneous fat, possibly containing thin septa [13]. Other cases have reported a heterogeneous mass with muscle and fat tissue [10, 12]. The ratio of the two tissues can vary on CT scanning according to the composition of each within the tumour. MRI may demonstrate the smooth muscle elements as areas of intermediate signal intensity on T1-weighted images. In large lesions, calcification may be present [14].

Macroscopically, the tumour is either partially or completely encapsulated and upon sectioning demonstrates a yellow/white cut surface [9]. Histologically, the tumour consists of a mix of mature adipose tissue and bundles or sheets of well-differentiated smooth muscle. The smooth muscle bundles are typically characterised by cytologically bland oval nuclei with eosinophilic fibrillar cytoplasm. The adipose component is entirely mature and lacks floret-like giant cells and lipoblasts [1]. The tumour lacks atypia and mitoses and displays little vascular proliferation [1, 15]. Bizarre, multilobulated nuclei have been reported within the myoid cells, this being considered an expression of a regressive phenomenon, as observed in the “bizarre” leiomyoma of the uterus [15]. The cells are immunoreactive for smooth muscle actin and desmin and may also contain oestrogen and progesterone receptors [16].

The differential diagnosis is important due to the number of lipomatous tumour variants [17]. Dedifferentiated liposarcoma, spindle cell lipoma, angiomyolipoma and leiomyoma with fatty degeneration must all be ruled out. Meis and Enzinger reported that deeply situated tumours were most likely to be confused with well-differentiated liposarcoma [1]. However, myolipoma differs from dedifferentiated liposarcoma due to the absence of atypical hyperchromatic nuclei in the fatty component and cytological atypia in the dedifferentiated component. Spindle cell lipoma is rare in the typical locations of myolipoma and is composed of bland spindle-shaped cells, lacking smooth muscle differentiation. Leiomyoma with fatty degeneration lacks the regular distribution of fat that is present in myolipoma. Angiomyolipoma has medium sized arteries with thick muscular walls as well as HMB-45 reactive epithelioid smooth muscle cells [9]. It is associated with tuberous sclerosis, whereas myolipoma is not. Furthermore, myolipoma is a benign tumour with no malignant potential whereas deep seated atypical lipoma has the potential to dedifferentiate into liposarcoma [18].

Surgical excision is the treatment of choice for these lesions. To date, there are no reports of recurrence and the complication rate is minimal.

This case represents an exceedingly rare and interesting benign tumour. Surgeons and histopathologists should be aware of the lesion in order to correctly diagnose and treat it. We report a new location for the tumour occurring in the youngest patient diagnosed so far. Despite the benign nature of the tumour, radiological investigations may indicate a more sinister diagnosis. Therefore, excisional biopsy in a specialized centre, where the necessary expertise is available, is recommended.
